# Intraperitoneal Administration of Silymarin Protects End Organs from Multivisceral Ischemia/Reperfusion Injury in a Rat Model

**DOI:** 10.5935/1678-9741.20160072

**Published:** 2016

**Authors:** Aydemir Koçarslan, Sezen Koçarslan, Mehmet Salih Aydin, Şamil Gunay, Mahmut Alp Karahan, Abdullah Taşkın, Murat Üstunel, Nurten Aksoy

**Affiliations:** 1 Kahramanmaraş Sütçü Imam University, Faculty of Medicine, Department of Cardiovascular Surgery, Kahramanmaraş, Turkey.; 2 Kahramanmaraş Sütçü Imam University, Faculty of Medicine, Department of Pathology, Kahramanmaraş, Turkey.; 3 Harran University, Faculty of Medicine, Department of Cardiovascular Surgery, Sanliurfa, Turkey.; 4 Harran University, Faculty of Medicine, Department of Thoracic Surgery, Sanliurfa, Turkey.; 5 Harran University, Faculty of Medicine, Department of Anesthesia and Reanimation, Sanliurfa, Turkey.; 6 Harran University, Faculty of Medicine, Department of Biochemistry, Sanliurfa, Turkey.; 7 Şanlıurfa Maternity Hospital, Department of Biochemistry, Sanliurfa, Turkey.

**Keywords:** Aorta/surgery, Reperfusion Injury, Silymarin, Rats, Wistar

## Abstract

**Objective:**

To determine whether intraperitoneal silymarin administration has favorable effects on the heart, lungs, kidney, and liver and on oxidative stress in a rat model of supraceliac aorta ischemia/reperfusion injury.

**Methods:**

Thirty male Wistar albino rats were divided equally into three groups: sham, control, and silymarin. The control and silymarin groups underwent supraceliac aortic occlusion for 45 min, followed by a 60 min period of reperfusion under terminal anesthesia. In the silymarin group, silymarin was administered intraperitoneally during ischemia at a dose of 200 mg/kg. Rats were euthanized using terminal anesthesia, and blood was collected from the inferior vena cava for total antioxidant capacity, total oxidative status, and oxidative stress index measurement. Lungs, heart, liver and kidney tissues were histologically examined.

**Results:**

Ischemia/reperfusion injury significantly increased histopathological damage as well as the total oxidative status and oxidative stress index levels in the blood samples. The silymarin group incurred significantly lesser damage to the lungs, liver and kidneys than the control group, while no differences were observed in the myocardium. Furthermore, the silymarin group had significantly lower total oxidative status and oxidative stress index levels than the control group.

**Conclusion:**

Intraperitoneal administration of silymarin reduces oxidative stress and protects the liver, kidney, and lungs from acute supraceliac abdominal aorta ischemia/reperfusion injury in the rat model.

**Table:** 

**Abbreviations, acronyms & symbols**	
I/R	=Ischemia-Reperfusion
OSI	=Oxidative stress index
TAC	=Total antioxidant capacity
TOS	=Total oxidative status

## INTRODUCTION

Acute supraceliac abdominal aorta ischemia may occur during treatment for abdominal aortic aneurysms, dissection repair of acute thromboembolism with aortic atherosclerosis, or trauma surgery. After reperfusion is performed, in such cases, reactive oxygen species are generated and there is excess production of pro-inflammatory molecules and a subsequent inflammatory response, which can lead to damage to internal organs, such as the lungs, liver, kidneys, intestines and heart, as well as death^[^^1^^]^. Multiple organ dysfunction occurring after abdominal aortic surgery is one of the major causes of mortality and morbidity, with 25% of the deaths that occur after elective abdominal aortic repair being related to multiple organ dysfunction^[^^2^^]^. Silymarin, which is a mixture of flavonoids and polyphenols, has been shown to exhibit a variety of pharmacological activities as well as antioxidant, anti-inflammatory, immunomodulatory, hepatoprotective, neuroprotective, renoprotective [against ischemia-reperfusion (I/R) injury], gastroprotective, antibacterial, antiviral, antithrombotic, and vasodilatory effects in many experimental and clinical studies^[^^3^^,^^4^^]^. It reduces oxidative damage by balancing the antioxidant status and regulating inflammatory mediators^[^^3^^]^. It has been reported that silymarin reduces I/R damage in the heart, lungs, kidneys and liver, but there are no studies on the preventive effects of silymarin in organs in the supraceliac aorta I/R model^[^^3^^-^^5^^]^. The aim of this study is to examine silymarin's protective effects on end organs (heart, lungs, liver and kidneys) in a rat model of acute supraceliac aorta I/R.

## METHODS

### Animals and Grouping

This experimental study was approved by the local ethics committee for animal experiments (Dollvet Veterinary Vaccines Biological Substance Drug Production Industry and Trade Inc. Şanlıurfa, Turkey). The experiments were conducted on 30 three-month-old Wistar albino rats that ranged in weight from 200 g to 250 g. All the animals were kept under standard conditions and were treated according to the guidelines of the National Institutes of Health. The animals were kept in a 12h light/dark cycle (lights were turned on at 6 am). The animals were deprived of food and water for 12h before surgery. The experiments were conducted at the Dollvet Laboratory of Experimental Research.

The rats were randomly divided into three experimental groups: sham (n=10), control (n=10) (I/R), and silymarin (n=10) (I/R-silymarin). They were anesthetized intraperitoneally with xylazine (10 mg/kg) and ketamine hydrochloride (50 mg/kg). The abdomens of the animals were shaved and then cleaned with a povidone-iodine solution, and an abdominal midline incision was made. In the sham group, only a laparotomy was carried out. In the control group, a cross clamp was placed on the supraceliac aorta and ischemia was applied for 45 min; this was followed by reperfusion for 60 min. In the silymarin group, the cross-clamp was placed on the supraceliac aorta for 45 min, and this was followed by reperfusion for 60 min. Silymarin was administered during ischemia at a dose of 200 mg/kg via the intraperitoneal route^[^^6^^]^. At the end of the study, blood samples were obtained from the inferior vena cava of the rats, and heart, lung, liver and kidney tissues were sampled and placed in formalin for pathological examination. The blood samples obtained were centrifuged, and plasma was separated and stored in the freezer at -80°C until biochemical analyses were performed. Silymarin tablets were obtained from the pharmacy (Sigma-Aldrich, St. Louis, MO, USA). A silymarin tablet of 85% purity was dissolved with 1% dimethyl sulfoxide before it was administered.

### Biochemical Tests

Serum levels of total oxidative status (TOS) and total antioxidant capacity (TAC) were assessed using a new automated colorimetric measurement method developed by Erel^[^^7^^]^, and calculated using the following formula: Oxidative stress index (OSI, arbitrary units) = TOS (nmol H_2_O_2_ equiv/mg protein)/TAC (nmol Trolox equiv/mg protein)^[^^8^^]^.

### Histopathological Evaluation

The hearts, lungs, kidneys, and livers of the animals were harvested and fixed in 10% formaldehyde solution. After the specimens were embedded in paraffin and 5 µm sections had been cut, they were stained with hematoxylin and eosin for light microscopic observation.

The prepared sections were examined under a light microscope at a magnification of ×20 (Olympus BX51 TF; Olympus, Melville, NY, USA). The samples were then histologically graded according to the severity of the injury by using a predetermined scoring system^[^^9^^-^^11^^]^. The histological indicators of I/R injury in the kidney evaluation were tubular necrosis, interstitial edema, loss of brush border, and cast formation. If there were no changes, a score of 0 was assigned; if there were medium or mild changes, the score assigned was 1; and if there were severe changes, the score was 2. The histological indicators of I/R injury in the lung evaluation were alveolar congestion, intra-alveolar hemorrhage, and interstitial perivascular infiltration of neutrophils. A score of 0 was assigned when those characteristics were absent; 1 was assigned for mild focal involvement; 2, for moderate focal involvement; and 3, for severely marked lung involvement. In the histological examination of the heart, interstitial edema, inflammatory cell infiltration, and coagulation necrosis were considered as indicators of I/R injury. A score of 0 was assigned when no changes were observed; 1 was assigned for slight focal involvement; 2, for medium focal involvement; and 3, for severely marked involvement. In the histopathological examination of the liver, nuclear pyknosis, necrosis, neutrophil infiltration, and loss of intercellular borders were evaluated. A score of 0 was assigned for minimal or no evidence of injury; grade 1, for mild injury consisting of focal nuclear pyknosis; grade 2, for moderate to severe injury, with extensive nuclear pyknosis and loss of intercellular borders; and grade 3, for severe necrosis, with disintegration of hepatic cords and neutrophil infiltration. The histopathological evaluation was conducted by the same pathologist in a blinded manner.

### Statistical Analysis

The data were analyzed using Statistical Package for the Social Sciences (SPSS for Windows 11.5, Chicago, IL, USA). Continuous variables were presented as mean ± standard deviation, and discrete variables were presented using their frequency or percentage distribution. The distribution of continuous variables was examined using a one-sample Kolmogorov-Smirnov test, and it was found that the data for all the groups were normally distributed. Therefore, non-parametric independent group comparisons were made. For multiple comparisons, a Kruskal-Wallis test was used, and for comparisons between groups, a Mann-Whitney test was used if any statistical significance was found. A two-sided *P* value of <0.05 was considered to indicate statistical significance.

## RESULTS

**Fig. 1:**
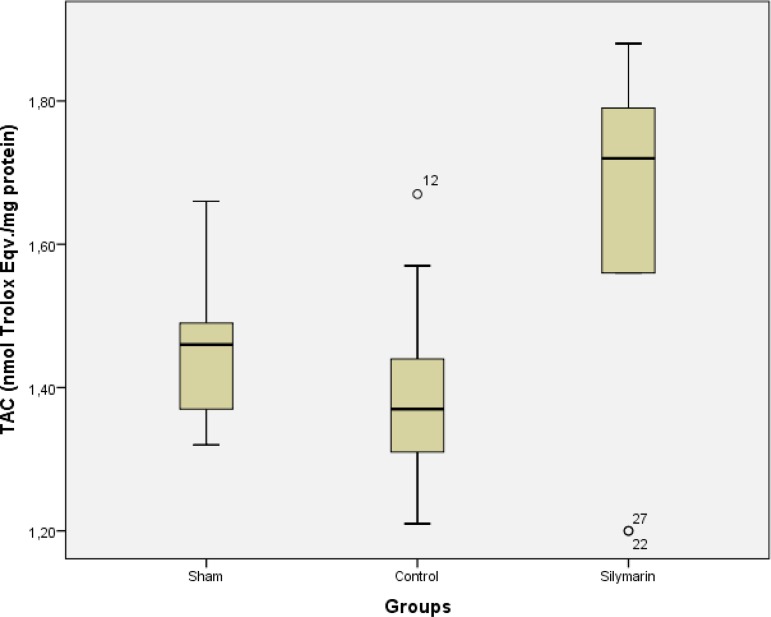
TAC levels for sham, control and silymarin groups. P<0.001 (for all comparisons) compared with sham and control groups

**Fig. 2:**
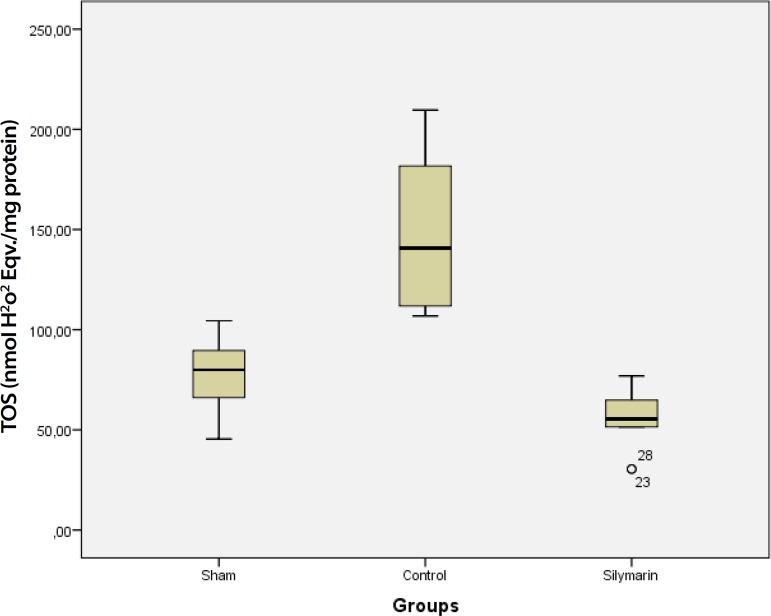
TOS levels in sham, control and silymarin groups. P<0.001 (for all comparisons) compared with sham and silymarin groups

**Fig. 3:**
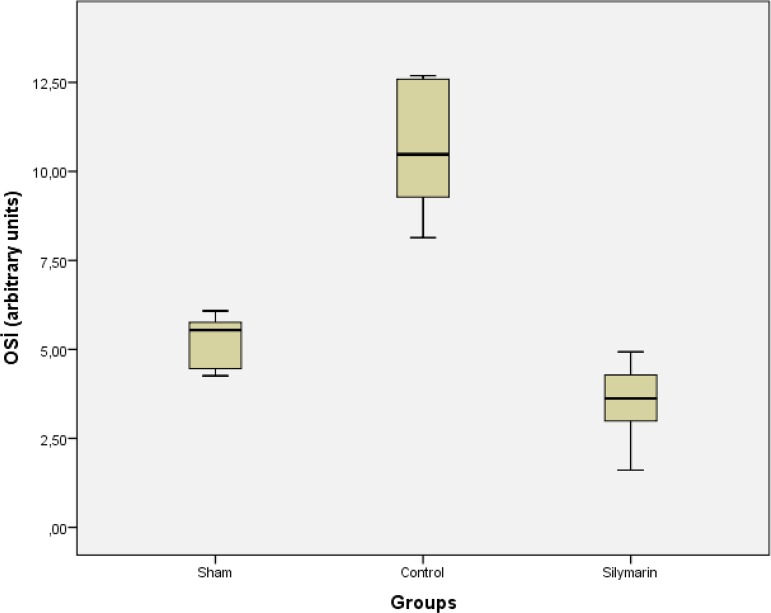
OSI levels in sham, control and silymarin groups. P<0.001 (for all comparisons) compared with sham and silymarin groups

**Fig. 4:**
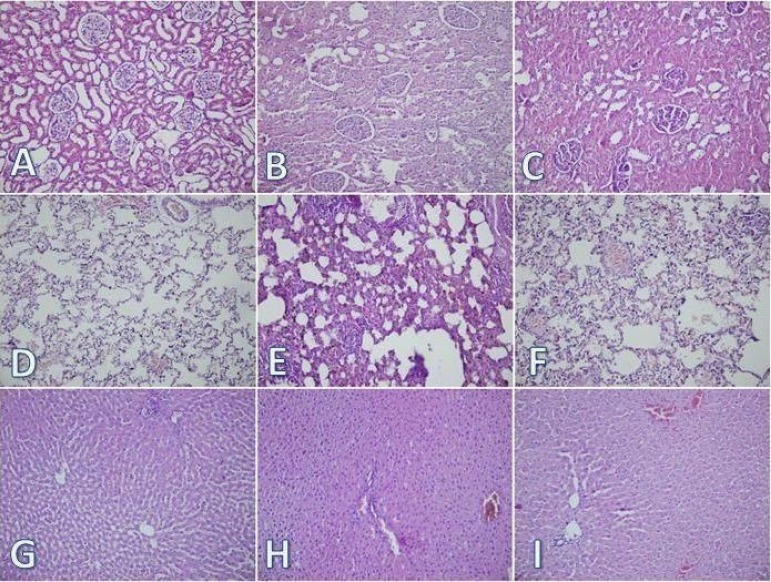
A, B, C are the kidney tissues of the sham, control and silymarin-treated groups, respectively. D, E, F are the lung tissues of the sham, control and silymarin-treated groups, respectively. G, H, I are the liver tissues of the sham, control and silymarin-treated groups, respectively.

All the animals were kept alive until the blood samples were taken. TAC activity was higher in blood samples from the silymarin group than in samples from the sham and control groups (*P*<0.001), but there was no statistically significant difference between the sham and the control groups (*P*>0.05) (Table 1, Figure 1). Furthermore, TOS activity and OSI of the control group were higher than in the sham and silymarin group (*P*<0.001 for all comparisons) (Table 1, Figures 2 and 3). The scores for tissue damage to the kidney, lungs, liver, and heart are presented in Table 1. The scores for kidney, lungs and liver were significantly higher in the control group than in the sham and silymarin groups (*P*<0.05). In the histopathological examination of the sham group, no changes were observed in the kidneys, lungs, liver (Figures 4A, 4D, and 4G) or heart. Histopathological examination of the control group revealed cast formation, brush border loss, and interstitial edema in the kidney; intra-alveolar hemorrhage, interstitial perivascular infiltration of neutrophils, and alveolar congestion in the lungs; and nuclear pyknosis, necrosis, neutrophil infiltration, and loss of intercellular borders in the liver (Figure 4B, 4E, and 4H). In the silymarin group, fewer histopathological changes were observed in the kidneys, lungs and livers than those in the control group (Figure 4C, 4F, and 4I). No significant difference was observed in the histopathological findings for the heart between all three groups under a light microscope.

**Table 1:** Sham, control and silymarin groups blood biochemistry and tissue histopathological parameters.

	**Sham Group**	**Control Group**	**Silymarin Group**	***P***
	**n=10**	**n=10**	**n=10**	**Kruskal-Wallis**
TAC	1.46±0.32	1.39±0.17	1.65±0.28*	<0.001
TOS	77.11+22.54	150.16±44.62+	55.81±17.25	<0.001
OSI	5.22±0.81	10.63±2.01+	3.48±1.27	<0.001
Lung	2.2±0.4	6.2±1.2+	2.8±0.6	<0.05
Kidney	2.8±0.5	5±0.9+	3±0.7	<0.05
Liver	2.8±0.4	9.2±2.1+	3.8±0.7	<0.05
Heart	1	1	1	>0.05

TAC=total antioxidant capacity; TOS=total oxidant status; OSI=oxidative stress index

*P*<0.05 was considered as statistically significant.

* *P*<0.001 (for all comparisons) compared with sham and control group.

+ *P*<0.001 (for all comparisons) compared with sham and silymarin group.

## DISCUSSION

In this experimental study, we observed the protective effects of silymarin in end organs, such as the lungs, liver and kidneys, against aortic supraceliac I/R injury. TOS and OSI values as well as histopathological damage scores were higher in the control group than in the sham and silymarin groups.

During open surgery of the abdominal aorta, the placement of a cross-clamp at the supraceliac level causes I/R damage to the bowel, kidneys, liver and lower limbs. I/R results in the transport of either bacteria or endotoxins across the intestinal mucosal barrier, leakage of reactive oxygen species and inflammatory cytokines into systemic circulation, kidney tubule damage, kidney failure, and acute damage to the lungs, liver and other internal organs^[^^12^^]^. It has been reported that various plant extracts have protective effects against I/R damage. Silymarin is one of them. Silymarin is derived from dry thistle seeds (*Silybum marianum*). Milk thistle is the oldest and best studied plant in the treatment of liver diseases, with the first records of its therapeutic effects being found in the Old Testament^[^^3^^]^. A standard silymarin dry extract is a bioflavonoid complex that consists of at least seven flavonolignans. The seven main components of silymarin are taxifolin, silychristin, silydianin, silybin A, silybin B, iso-silybin A, and iso-silybin B. Taxifolin is the only isomer in the silymarin extract that is not a flavonolignan and has strong antioxidant activity^[^^3^^]^. Silybin is the main component of this complex extract, accounting for 60-70% of the constituents; it is a potent inhibitor of nuclear factor kappa B (NF-kB) activation, and it is induced by a variety of anti-inflammatory agents^[^^13^^]^. It has been reported that the anti-inflammatory and anti-cancer effects of silymarin and other flavonolignans are related to their potency inhibition of NF-kB. This transcription factor is involved in the arrangement of several genes associated with immune function, inflammation stress response, cell differentiation, apoptosis and cell survival, which are required for cancer development and progression^[^^14^^]^. Silymarin superoxide ions act in a similar manner to hydroxyl radicals, hypochlorous acid, and singlet molecular oxygen collectors^[^^15^^]^. It increases the level of cellular glutathione and reduces tumor promoter activity^[^^16^^]^. In addition, silymarin inhibits lipid peroxidation and reduces free radicaldependent tissue damage^[^^17^^]^. It has also been shown that silymarin increases the activity of antioxidant enzymes, such as superoxide dismutase, glutathione peroxidase and catalase, in the pancreas of rats^[^^18^^]^.

Rao & Viswanath^[^^19^^]^ found that oral silymarin use as phytomedicine has cardioprotective properties against I/R-induced myocardial infarction in rats, and the myocardial protective effects of silymarin have also been demonstrated in cardiac injury induced by anthracycline, cisplatin, doxorubicin, adriamycin, and sodium fluoride in rats^[^^3^^]^. In our study, the heart I/R injury associated with distant organ damage could not be observed under a light microscope; this is probably related to the short retention time of reperfusion. However, Altaei et al.^[^^20^^]^ have shown that oral silymarin treatment before surgery protects the heart against reperfusion injury and inflammation through anti-inflammatory and antioxidant activity during CABG surgery in humans.

Several studies have reported the protective effects of silymarin in the lungs in different situations and via different mechanisms. Jin et al.^[^^21^^]^ showed that oral silymarin treatment was able to improve pulmonary vascular dysfunction following lung I/R injury via the HIF-1α-iNOS pathway. Sharma et al.^[^^22^^]^ showed that silibinin significantly stimulates growth inhibition, moderate cell-cycle arrest and apoptotic death in both small-cell and non-small cell human lung carcinoma cells. The pulmonary protective effects of silymarin against I/R injury via intraperitoneal route have been shown in this study for the first time. The renoprotective effects of silymarin have also been discussed in several studies, including in nephrotoxic drugs in rats^[^^23^^-^^25^^]^. Oral silymarin use has also been studied in different kidney I/R models, and is reported to have favorable effects with different potential mechanisms, including primarily antioxidant mechanisms^[^^26^^,^^27^^]^. Likewise, in our study, the histopathological findings showed that intraperitoneal silymarin use has renoprotective effects against I/R injury.

Several studies have shown that silymarin has protective effects against I/R injury to the liver, and that silymarin can also protect the liver against hepatotoxic drugs and liver cancer^[^^28^^]^. In a study by Ligeret et al.^[^^29^^]^, it was reported that a preservation solution with silibinin has protective effects against cold preservation-warm reperfusion injury on experimental liver transplantation. Additionally, Wu et al.^[^^30^^]^ showed that intravenous silymarin use protects liver against I/R injury. Our study has also provided histopathological evidence for the protection of the liver against I/R injury by intraperitoneal administration of silymarin. Thus, in this study, we observed the protective effects of silymarin in the lungs, kidneys and liver through histopathological evaluation. The significant oxidative stress in the control group when compared to the sham and silymarin groups also emphasizes the antioxidant properties of silymarin, which may serve as a protective mechanism in the rat acute supraceliac abdominal aorta I/R model.

We believe that there are sufficient preclinical research findings about the molecular antioxidant, anti-inflammatory, and anti-cancer effects of silymarin, as well as its drug toxicity, bioavailability, pharmacokinetics, and novel drug delivery approaches^[^^3^^]^. Still, the clinical implications and appropriate pathophysiological mechanisms underlying its effects need to be elucidated in large-scale clinical studies.

The limitations of this study are as follows: the effect of oral intake of silymarin in rats has not been evaluated. Another limitation is that the biochemical markers urea, creatinine, creatinine phosphokinase, creatinine kinase-myocardial band, aspartate aminotransferase, and alanine aminotransferase have not been examined. Moreover, the effects of silymarin on the intestines, the spinal cord, and the brain have not been studied either.

## CONCLUSION

In conclusion, silymarin has cardioprotective, renoprotective, neuroprotective, pulmonary protective, anti-inflammatory, antioxidant, immunomodulatory, anti-proliferative, hepatoprotective and antidiabetic effects that have been demonstrated in clinical and experimental studies. Despite our limitations, this study has shown that the administration of silymarin through the intraperitoneal route reduces histopathological damage to the liver, kidneys and lungs, as well as oxidative stress in the I/R model of the supraceliac abdominal aorta for the first time. The findings indicate that silymarin treatment may reduce tissue damage and OSI. They therefore lay the foundation for future clinical trials on the efficacy of silymarin.

**Table:** 

**Authors' roles & responsibilities**	
AK	Analysis and /or interpretation of data, statistical analysis, final approval of the manuscript, conception and study design, conduct of procedures and/or experiments, writing of the manuscript or review of its content
SK	Analysis and/or interpretation of data, final approval of manuscript
MSA	Statistical analysis, conception and study design, conduct of procedures and/or experiments
ŞG	Conduct of procedures and/or experiments, writing of the manuscript or review of its content
MAK	Statistical analysis, final approval of manuscript
AT	Statistical analysis, final approval of the manuscript, conception and study design, conduct of procedures and/ or experiments
MU	Statistical analysis, final approval of the manuscript, writing of the manuscript or review of its content
NA	Writing of the manuscript or review of its content
